# Supplementary material for: Predict the incidence of Guillain Barré Syndrome and arbovirus infection in Mexico, 2014–2019
Source: PLOS Glob Public Health. 2022 Mar 25;2(3):e0000137. doi: 10.1371/journal.pgph.0000137 (PMC10022261; doi:10.1371/journal.pgph.0000137)
Supplement: Attachment — Submitted filename: response to reviews 301121.docx [file pgph.0000137.s001.docx]

Temporal Correlation Between Guillain Barré Syndrome and Arbovirus Infection in Mexico, 2014-2019.

'Response to Reviewers'.

First, we want to thank the reviewers for their comments and observations that are very valuable to improve the manuscript.

We have answered each of your observations as clearly as possible, below we list each one:

Reviewer #1

Comment: The main objective of this paper is predict the incidence of Guillain-Barré Syndrome in Mexico from the incidence of Dengue, Zika and Chikungunya diseases using a linear regression model applied to national surveillance data collected between 2014 and 2019. These three arboviroses already represent a heavy burden for the population of Latin America, GBS is an added complication for both patients and health systems. The data and analysis presented in this paper is of relevance to a public health audience.

* Title and abstract
Comment: The title of the paper and the objective section of the abstract do not reflect the main objective of the study which is to predict the incidence of GBS, of public health interest, whereas correlations appear secondary when reading the whole manuscript.

Answer: We have adjusted the title and in the abstract to the purpose of the manuscript: Predict the incidence of Guillain Barré Syndrome and Arbovirus Infection in Mexico, 2014-2019.

Comment: The methods section of the abstract mentions a five-year analysis when a six-year analysis is presented in the paper (2014-2019).

Answer: we have made the change to six years.

Comment: In the results section: “The increase of incidence of GBS in Mexico is correlated with the epidemic years of DENV and ZIKV”. None of the results of the paper supports this particular claim. The linear regression or the correlations shown in the paper do not allow drawing conclusions that distinguish epidemic vs. non-epidemic years.

Answer: You are right, we do not distinguish between epidemic vs non epidemic year.

The new sentence says: The increase in the incidence of GBS in Mexico is positive correlated with the epidemic years of DENV and ZIKV. For every 1,000 cases of dengue, 2 cases of GBS occurred, and for every 1,000 cases of Zika, 3 cases of GBS occurred. Not significant correlation between GBS CHIKV cases was found.

* Background

Comment: This introduction does not mention the different etiologies of GBS other than arboviruses (eg: Campylobacter infections). Surprisingly, the lethality figure mentioned in the abstract is not found in the background section; with a proper reference, this figure would help stressing out the importance of GBS as a health problem.

Answer:

In the introduction we add the next sentence: About 20% of patients with GBS develop respiratory failure and require mechanical ventilation. The mortality rate is estimated at 3–10% for patients with GBS even with the best medical care available. But the impact on patients' life which may go beyond their residual disability or impairment almost 30% of them had to make substantial changes in daily life.

The underlying causes of the GBS pathology are not yet fully understood, However, the following factors have been described as a trigger for GBS: Many infections have been linked with GBS. The most common are gastrointestinal or respiratory illnesses. Up to 70% of patients have reported an antecedent illness in the 1 to 6 weeks before the presentation of GBS, about a third of all cases of GBS are preceded by Campylobacter jejuni infection.

Viral infection like Haemophilus influenzae, influenza virus, Epstein–Barr virus, cytomegalovirus, human immunodeficiency virus, Hepatitis E virus and SARS-COV-2 are linked for GBS; also several neurological manifestations, including GBS, are associated with ZIKV, DENV or CHIKV.

Comment: Line 73: The formulation of the objective is too vague. In my opinion the interest of the paper is the presentation of a predictive model and should be focused on it. This should also be reflected in the title of the paper.

Answer: we had change the title and in line 73 we change for the next sentence: “The objective of this study is to present a predictive model of GBS cases from DENV, CHIKV and ZIKV simultaneous circulating arboviruses in Mexico from 2014 to 2019…”

* Methods

Comment: Linear regression: Line 127 “to correlate”; the term should be changed. The objective of the model is not to study correlations but to predict the number of GBS cases for each increase in the incidence of ZKV/DENV/CHIKV. Please precise that the dependent variable is also the weekly number of GBS cases (line 127).

Answer: We changed “to correlate” for “to predict”.

Yes, the dependent variable was the **weekly** number of GBS cases, we had precise that.

Comment: Please also clarify what is the date used for the cases: date of symptom onset, date of reporting?

Answer: The data used for the cases was for date of symptoms onset. In line 146 we made the clarify.

Comment: It is unclear what variables were compared in the ANOVA: “weekly means and annual GBS cases”; is the variance of weekly cases of GBS compared between each year? Why?

Answer: Yes, it is unclear, we did an analysis of variance was performed between the weekly means by year of number GBS cases. We changed the for median analysis. We do this, trying to see if there was a difference in the weekly median of GBS between years because arbovirus (DENV, ZIKV and CHIKV) they are not the same impact by year.

* Results

Table 1

Comment: Please harmonize acronyms in Table 1 with the rest of the text: SGB vs GBS, DEN vs DENV, etc. The first year should be 2014 and not 2015.

Answer: we had made the change.

Comment: There is clearly a change in the yearly number of cases of GBS before and after 2016. As mentioned in the discussion, this could be attributed to a change in the reporting policy especially in the context of ZKV epidemic and international awareness. This could be estimated with an interrupted time series analysis (ITSA, segmented linear regression) by adding to the linear regression model a step variable (0 before 2016, 1 after) to model this change in the surveillance system. The associated coefficient would estimate the before/after variation.

Answer: we did an interrupted time series analysis was carried out through a segmented linear regression, The estimation of the notification GBS cases before/after 2016 was a 3.048, sig. 0.000

Comment: I find a lack of epidemiological rigor in the way the evolution of yearly cases of GBS and arboviruses cases is presented. Especially: line 146: “the incidence and number of DEN cases doubled from 2015 and 2019” is quite misleading. Graph 1 clearly shows that each year Mexico experiences a DENV epidemic, the magnitude of which appears to decrease slightly between 2014 and 2018. Only in 2019 the epidemic was particularly big. Do the authors have any explanation for this observation?

Answer: We had made the change for the next sentence: The cases and incidence of dengue per year presented changes both of increase and decrease, in 2019 there was an outbreak of dengue in Mexico,the behaviour of dengue fever in Mexico over the years is dynamic, and the outbreak patterns coincide with those present at the regional level, among the factors involved is the phenomenon of global warming, which expands the habitat of the vector, the poor treatment of bodies of water that favors their growth and increases in population density, migratory phenomena and human movements, which together increase the transmission possibilities.

Table2

Comment: Table2. The ANOVA shows that the yearly mean of weekly GBS cases are statistically different over the period, this result in itself is not much more informative than those shown in Table 1 and Graph 1. It clearly shows an increase between 2014-2015 and 2016-2019; another argument in favor of a change of reporting policy.

Answer: We changed the for median analysis. We do this, trying to see if there was a difference in the weekly median of GBS cases between years because arbovirus (DENV, ZIKV and CHIKV) they are not the same impact by year.

Comment: Line 168: why is this statement important? For a linear regression, only the distribution of error terms should be normal. Was this checked?

Anwer: We removed that phrase, You're right. We had described it to do the median analysis. Not for regression analysis. If we check the errors.

Coment: Line 163/Line 171: please be clearer about why despite positive correlations between independent variables only limited collinearity was found. The correlations presented in the appendix do not bring much added information.

Answer: We performed the tolerance analysis to comply with the assumption of the linear regressions that the independent variables in this case DENV, CHIV and ZIKV. finding that there is multicollinearity but sufficient to perform the linear regression using the value of the variance inflation factor (VIF).

Also the condition index in the collinearity analysis is between 1 -10 which allows us to perform the linear regression.

In the appendix we have added the inflation index of the variance.

Comment: Table 3 The values shown for the coefficients and associated 95%CI have too few significant numbers. Maybe a change in the unit of the independent variables (i.e.: 1000 cases instead of single case) would make the table and the equation more readable. It would also better fit the interpretation given in the discussion (lines 187 and 188).

Answer: We had made the change using the indepent variables by 1000 cases.

Comment: Have you tried a simpler model using a single independent variable grouping all cases of arboviruses instead of distinguishing the diseases?

Answer: Yes, we performed the analysis gathering all the arbovirus cases, finding an R squared value = 0.098 and a B value = 0.001 with p value = 0.000, as the R value was lower, we preferred to keep the model separately from the independent variables.

In the repository database we add the variable arbovirus.

Comment: Again, I suggest trying an ITSA to explore the effect of change of policy in the reporting of GBS.

Answer: We appreciate your suggestion, we found that using ITSA after 2016 explains a 3-fold increase in the notification of GBS cases.

Comment: * Discussion The constant of the model (4.2 to 5.1 cases of GBS per week in absence of arboviroses) should be discussed. So should the negative coefficient for CHIKV (cf lines 216-218).

Answer: We have described the following in the discusión “Our model it indicates that there are 2.5 cases of GBS per week, they do not correlate to arboviruses, this indicates how it is known that there are different etiologies of GBS, probably the most studied infection by Campylobacter jejuni infection”

Comment: Line 188: “This finding is very similar”, the ZIKV-associated incidence of GBS estimated in this article is 10 times greater than the mean value found in ref #21. I don’t think ‘very similar’ is appropriate.

Answer: Yes you are right, we change the paragraph to “In our result the ZIKV-associated incidence of GBS estimated is 10 times greater to that published in an ecological study on the American continent, where 2(0.5 - 4.5) GBS cases were estimated for every 10,000 ZIKV cases; Nevertheless, the frequency of reported suspect Zika cases varied substantially and was highly uncertain in that study”.

Comment: Lines 225-228: This is unclear. What is meant by a “temporality bias”? Indeed a time lag exists between ZIKV infection and GBS onset: was it taken into account into the analysis? If not, why?

Answer: Yes it was unclear, we want to said that for the study design, the bradford and hill criteria of temporality cannot be measured. Indeed, there is a lag between arbovirus infection and GBS, in this case it cannot be considered since it was not measured individually, we write it as follows:

The limitations of the study are as follows. 1) The study was not designed to measure a causal relationship; Due to the nature of the data, an exposure-disease relationship cannot be assumed, that the exposure must precede the onset of the disease (53).

Either analyze demographic factors, such as the age or sex of the patients, although it has been shown that older patients and males have a higher incidence of GBS. 2) This is an ecological study, so its interpretation should not be at the individual or association level so as not to fall into an ecological fallacy.

Comment: Lines 235-236: There is a clear shift before and after 2016 in the total number of GBS cases reported each year, despite the absence of Zika epidemic after 2016. A change in surveillance and reporting of GBS is a major bias and should be explored before discussing trend evolution between 2014 and 2019.

Answer: We have specified how the change in epidemiological surveillance occurred, both in the methodology and in the discussion. Lines 94-96 and 280-283.

Comment: Line 233: “infected with GBS”: this phrasing is not appropriate; GBS is an auto-immune disorder that may result from an infection, but not an infectious disease in itself. Did the authors write GBS instead of ZKV?

Answer: Yes infected with GBS is not correct we want to said ZIKV, anyway we eliminate the phrase since when rereading it does not contribute much.

Comment: Line 238: “DENV” should be replaced by “GBS”

Answer: Yes we had made the replaced.

Reviewer #2: Cumulative data support an association between ZIKV and GBS in some parts of the world. However, uncertainty remains regarding the association between other arbovirus cases and GBS.

The authors conducted a population-based study analysing a 6-year time series in Mexico (2014-2019). They assessed the correlation between Guillain-Barré syndrome cases and arboviruses cases (DENV, ZIKV, CHIKV).

The data are based on a nationwide epidemiological surveillance system of both GBS and arbovirus cases.

The methods are well explained and the results are clearly presented. The identification of arbovirus cases is well explained and clear. The gathering of data is well designed.

The authors conclude that GBS cases are positively correlated with DENV and ZIKV cases and determined the strenght of this association. On the opposite they report a negative correlation between CHIKV and GBS cases which is a matter of debate.

The authors also discuss thoroughly the limitations of their study.

The data seem robust to support the conclusions of the authors.

The manuscript is quite concise and clear and as a significant potential for publication.

However I have some remarks regarding the manuscript.

Comment; First, the statistical analysis plan need to be assessed by a times series analysis specialist to check if.

Answer: We have verified the analysis of the time series, performing the linear regression adding the segmentation to the analysis. We have detailed each point of the analysis of the time series in addition to making the database available to readers.

Comment: Second, the authors should strengthen what this study adds to the current knowledge about the subject. I suggest the introduction section should state more clearly what is already known about it and what remains to be studied.

Answer: we have changed the entire introduction attending your comment.

Comment: Even though the manuscrit is in general comprehensive some language editing should made throughout the manuscript to gain in clarity (ex l 94-95, 118, 121-122, l 204).

Answer:We have edited almost the entire manuscript for clarity as you indicate.

Comment: Some of the results are given in the discussion section(see L 188-189) (or the abstract "For every 1,000 cases of dengue, 2 cases of GBS occurred, and for every 1,000 cases of Zika, 3 cases of GBS occurred") and should be cited in the results section because it corresponds to the main objective of the study.

Answer: We have changed to the results section.

Comment: L 231 231 the authors state that "there is no evidence that coinfection between DENV and ZIKV increases the risk of GBS". This point has been assessed somewhere else and should be discussed see Leonhard et al <https://doi.org/10.1016/j.jns.2020.117272>

Answer: We have changed the wording of this paragraph, the article you mention suggests greater severity in GBS but no greater probability of developing GBS.

The new parragraf say “Note that coinfection between arboviruses was not analyzed, given that arbovirus cases were analyzed independently of each other; However, it is not yet known if the coinfection impacts clinical disease (54), but it has been described that GBS patients with laboratory evidence of both a recent ZIKV and CHIKV infection had a longer duration of hospitalization, were admitted to the ICU, and intubated significantly more frequently than the other patients with GBS (21)”

Comment: The authors find a negative correlation between CHIK and GBS cases. Tis point is not really discussed after. It could be interesting because some authors found a possible association between CHIKV and GBS (see also Leonhard et al).

Answer: We had discussed the negative correlation between CHIKV an GBS cases,

“studies have related the presence of IgM antibodies to CHIKV with GBS; however, it has been difficult to quantitatively estimate the incidence of CHIKV infection with neurological diseases. In the present study, no positive relationship was detected between CHIKV infection and GBS; The study of the relationship between chikungunya and GBS has been complex, since the populations where it has been studied exist endemic circulation of DENV, ZIKV and CHIKV the use of IgM and IgG antibodies reflects a previous infection with CHIKV, complexity resulting from their transmission dynamics, more integrative studies are needed investigating a combination of ZIKV, DENV, and CHIKV viruses and using a variety of approaches to answer questions related to the risk posed by these arboviruses. In our study we include symptomatic cases of CHIKV, asymptomatic cases can be at least 47% more than those included in this study. In the studies that have used PCR to detect CHIKV infection, they have had a low number of samples to associate CHIKV and GBS”

Comment: The time bias evoked by the authors should be discussed more thoroughly. Indeed this point limits the interpretation of the temporal correlation found here. Is the short timelapse (6-11 days) cited by the authors between arbovirus infection and GBS occurrence is really that certain?

Answer: we have changed the wording to be as follows The limitations of the study are as follows. 1) The study was not designed to measure a causal relationship; Due to the nature of the data, an exposure-disease relationship cannot be assumed, that the exposure must precede the onset of the disease.This is an ecological study, so its interpretation should not be at the individual or association level so as not to fall into an ecological fallacy.

Comment: The discussion section could be somewhat reviewed to clarify the authors’ point.
L 211 Do the authors refer to their result or to the literature?

Answer: we refer to the result of the study, “In our results we have estimated that for every 1,000 cases of DENV, 1.45 case of GBS occurs”.

Comment: Table 2 Do the authors refer to ANOVA of weekly means?

Answer: Yes,but we but we eliminate after attending the reviewer's comments 1

In the abstract the authors wrote 5-year time series whereas it is 6-year time series.

Answer: Yes, we are sorry for the mistake.

The authors
