# Supplementary material for: Predict the incidence of Guillain Barré Syndrome and arbovirus infection in Mexico, 2014–2019
Source: PLOS Glob Public Health. 2022 Mar 25;2(3):e0000137. doi: 10.1371/journal.pgph.0000137 (PMC10022261; doi:10.1371/journal.pgph.0000137)
Supplement: Attachment — Submitted filename: response to reviews 220222.docx [file pgph.0000137.s002.docx]

PGPH-D-21-00407R1
Predict the incidence of Guillain Barré Syndrome and Arbovirus infection in Mexico, 2014-2019
PLOS Global Public Health

'Response to Reviewers'.

First, we want to thank the reviewers for their comments and observations that are very valuable to improve the manuscript.

We have answered each of your observations as clearly as possible, below we list each one:

Reviewer #1: 


Specific comments:
- Graph 2 displays the same information as Graph 1 with a scale change and highlight of 2016 policy change. I suggest combining the two figures, especially given Graph 3 displays actual vs. modelled data.
- Line 211: the effect of 2016 policy change is missing from the equation of the model
- Line 217: the interpretation is not correct; after 2016 the notification increases by three (additional model) but is not multiplied by 3. The constant is 2.5 cases/week before 2016 and 5.5 cases/week after 2016.
- I suggest avoiding reporting zero p-values (i.e. p=0.000 in Table 1) and using p<0.0001 instead.

Answer: We have combined Figures 1 and 2 , we decided to emphasize in Figure 1 on the 2016 in GBS notification policy. since as you mention Figure 3 (now 2) shows the model data.

- We have clarified the effect in the model that the policy has on the notification of GBS cases after 2016, uses two equations for a better understanding.
- the interpretation of the model results has been corrected as you mentioned, an apology for the error.
- We have used the value of p as you suggest.

Reviewer #2:  A language editing remains necessary to gain in clarity in many parts of the manuscript considering some authors' points are still hard to catch (ex l223-226, 264-270).

Answer: We have revised the entire manuscript to make it more understandable, improving the writing and editing of almost the entire manuscript.

The authors
